# The Professional Culture of Community Pharmacy and the Provision of MTM Services

**DOI:** 10.3390/pharmacy6020025

**Published:** 2018-03-21

**Authors:** Meagen M. Rosenthal, Erin R. Holmes

**Affiliations:** Department of Pharmacy Administration, School of Pharmacy, University of Mississippi, 223 Faser Hall, University, MS 38677-1848, USA; erholmes@olemiss.edu

**Keywords:** professional culture, community pharmacy, advanced pharmacy services

## Abstract

The integration of advanced pharmacy services into community pharmacy practice is not complete. According to implementation research understanding professional culture, as a part of context, may provide insights for accelerating this process. There are three objectives in this study. The first objective of this study was to validate an adapted version of an organizational culture measure in a sample of United States’ (US) community pharmacists. The second objective was to examine potential relationships between the cultural factors identified using the validated instrument and a number of socialization and education variables. The third objective was to examine any relationships between the scores on the identified cultural factors and the provision of MTM services. This study was a cross-sectional online survey for community pharmacists in the southeastern US. The survey contained questions on socialization/education, respondents’ self-reported provision of medication therapy management (MTM) services, and the organizational culture profile (OCP). Analyses included descriptive statistics, a principle components analysis (PCA), independent samples *t*-test, and multivariate ordinal regression. A total of 303 surveys were completed. The PCA revealed a six-factor structure: social responsibility, innovation, people orientation, competitiveness, attention to detail, and reward orientation. Further analysis revealed significant relationships between social responsibility and years in practice, and people orientation and attention to detail and pharmacists’ training and practice setting. Significant positive relationships were observed between social responsibility, innovation, and competitiveness and the increased provision of MTM services. The significant relationships identified between the OCP factors and community pharmacist respondents’ provision of MTM services provides an important starting point for developing interventions to improve the uptake of practice change opportunities.

## 1. Background

As quality measures permeate pharmacy practice, and pharmacists are afforded new legislated opportunities to provide clinical services, it is integral that all members of the pharmacy profession are ready, and able, to adapt to these changes [1]. Fortunately, evidence of pharmacists’ clinical efficacy in helping to manage chronic conditions, such as diabetes, has been well documented [2]. In fact, there is even a growing body of evidence suggesting that pharmacists’ independent prescribing is a safe and effective way to help patients with chronic conditions such as hypertension and hyperlipidemia to meet guideline targets [3,4,5]. Unfortunately, these practices have not spread to all members of the pharmacy profession, and by extension to patients. 

For example, despite expressing interest in providing advanced pharmacy services such as medication therapy management (MTM) to patients, community pharmacists continue to struggle with a number of barriers including the lack of payment structures, support, and time [6]. Furthermore, pharmacists’ lack of time to provide these services results in patients not seeking pharmacy services, and reinforces pharmacists’ notion that patients do not want these services from pharmacists [7]. Some of these barriers are being addressed through national legislative efforts such as the “Pharmacy and Medically Underserved Areas Enhancement Act” (H.R.592), which, if passed, will allow pharmacists to seek reimbursement for the provision of clinical services [8]. While these fixes are incredibly important, alone they fail to address the complete context of community pharmacy practice. 

Implementation research has found that merely providing healthcare professionals, like pharmacists, with clinical evidence in support of patient services is not enough to ensure its successful application by clinicians [9,10]. Research in this field suggests that the “context” in which the clinician finds themselves plays an integral role in the implementation process [10]. Context, in this case, includes aspects of the social, economic, political, legal, physical, and cultural environments into which a change is being proposed [10]. Therefore, access to reimbursement only addresses the legal, economic, and social aspects of pharmacists’ context respectively. This study focuses on the potential impact of professional culture on the provision of medication therapy management (MTM) by community pharmacists.

Professional cultures are groups of people who share a pattern of values, beliefs, norms, and interpretations [11]. These values, beliefs, norms, and interpretations are developed through socialization and education processes [12]. The presence and importance of professional culture has been recognized by a number of authors [11,13,14]. Importantly, these authors also acknowledge that professional cultures exist within larger organizational cultures [11,15]. Preliminary work on professional culture has been conducted within healthcare settings examining the professional cultures of medicine, nursing, social work and pharmacy [16,17,18,19,20]. Some recent work has also established preliminary connections between various professional cultures’ approaches to learning, the definitions of credibility and constructiveness, and how feedback was integrated into the various practices of these professions [14].

However, much of the previous work on professional culture in healthcare settings has been observational in nature, seeking to characterize the culture, rather than measuring and linking it with professional behaviors or actions. Recent work with pharmacists in Canada has uncovered a number of relationships between pharmacy’s professional culture and the provision of clinical services by pharmacists [21]. In particular, respondents who saw greater value in the cultural factors innovation and competitiveness also provided an increased number of advanced pharmacy services such as medication reviews and immunizations [21]. Currently, there is limited research empirically examining the influence of professional culture of pharmacy on community pharmacists’ behaviors in the United States (US). The purpose of this work is to begin to characterize the professional culture of community pharmacists, and to examine potential relationships between the factors of professional culture, socialization and education, and the provision of MTM services. 

## 2. Objectives

There are three objectives in this study. The first objective of this study was to validate an adapted version of an organizational culture measure in a sample of US community pharmacists. The second objective was to examine potential relationships between the cultural factors identified using the validated instrument and a number of socialization and education variables. The third objective was to examine any relationships between the scores on the identified cultural factors and the provision of MTM services. 

## 3. Materials and Methods

*Study design*: A cross-sectional online survey was used for the study. This study was approved as exempt under the University of Mississippi Institutional Review Board (protocol #15x-027).

*Sample*: The sample was comprised of community pharmacists from the southeastern US. The focus on community pharmacists was primarily based on potential differences between the professional cultures of community and hospital pharmacist given their distinct practice settings. The second reason for the focus on community pharmacy was based on the fact that most pharmacists in the US work in the community setting, meaning that any insights gained from this work would be applicable to a large proportion of the pharmacist population [22]. The target sample size for this study is 300, and is based on an examination of the literature, which states that this is the minimum threshold required to validate a survey instrument [23]. 

The sample was collected with the assistance of a health research and marketing firm called DMD Healthcare Research (http://deltamarketingdynamics.com). This company has access to a large, nationally representative, database of community pharmacist panelists. The advantage of approaching this company to complete data collection was a guarantee to achieve the sample size.

*Procedure/data collection*: Each of the 1457 randomly selected potential community pharmacist participants received an invitation email through DMD Healthcare Research which contained the survey link. The survey was administered in February of 2016, and data collection was completed within that month. A small monetary incentive was provided for each completed survey. The online survey was developed through Qualtrics Inc. online software system (Provo, UT, USA). This software houses all anonymous survey responses on its own secure server, which can be accessed through the license maintained by the University of Mississippi. 

*Survey*: The online survey contained two sections. In the first section, the socialization and education variables and respondents’ self-reported provision of MTM services were captured (see Supplement 1 for complete survey). The socialization and education variables were culled from the definition of professional culture and were chosen to capture community pharmacists’ common experiences in becoming a pharmacist [12]. In particular, questions about how long the respondent had been in practice, his/her educational background, and current practice setting were asked. There were three of these questions. The questions for the provision of MTM services were generated through an examination of the definition of MTM offered by the American Pharmacists Association [24]. Questions in this survey focused on the number of immunizations, medication reviews, and disease management consultations provided in the previous month. These services were chosen because they correlate with measures used previously in the examination of Canadian pharmacists’ professional culture and provision of advanced pharmacy services [21]. Three questions focused specifically on the number of each of the MTM services provided.

The second section of the survey contained the OCP. The OCP was originally designed to measure person-organization fit through an evaluation of overlapping cultural values [25]. In particular, the instrument was administered to employees to evaluate personal cultural values and then to managers of an organization to evaluate the organization’s cultural values. The results of employees and managers were then compared to determine whether or not employee values matched with those of the organization [25]. This study was also replicated examining the fit between the culture of a hospital in Belgium and the culture of nursing staff working within the hospital [26]. Given the previous applications of this instrument evaluating individual, professional, and organizational cultures it was determined that an adapted version of the OCP could be applied to a preliminary evaluation of community pharmacists’ professional culture in this study.

In this survey the instrument was adapted to begin with a statement prompting the respondent to consider, “To what extent pharmacy is recognized for its…”, from “To what extent is your organization recognized for its…” [25]. Some examples of the 40 items of the OCP include adaptability, being innovative, being analytical, fairness, being results oriented, and being calm. All items were measured in a 5-point linear numeric scale from “not at all recognized” to “very much recognized”. A previous validation of these items revealed seven cultural factors: innovation, supportiveness, social responsibility, competitiveness, stability, performance orientation, and reward orientation [25]. 

Unlike other culture measurement tools, reliability analyses of the previously identified cultural factors of the OCP have been undertaken [25]. The reliability scores for factors identified using the instrument have ranged between 0.77 and 0.88, depending upon the population under study [25]. Previous work using the OCP from Canada and identified five cultural factors: supportiveness, social responsibility, competitiveness, performance orientation, and reward orientation [21]. Reliability scores for these factors ranged from 0.92 for supportiveness, to 0.76 for performance orientation [21]. This survey was not pilot tested, however, as mentioned all survey questioned mirrored those administered in a previous survey conducted in Canada, and face validity tests were performed with experts in the field [21].

*Analysis*: All analyses were conducted using SPSS for Macintosh, version 22.0. An initial examination of the data from all MTM service variables revealed widely ranging mean and median scores (i.e., medication reviews mean = 19.09, median = 2.50; immunizations mean = 17.75, median = 10.00; disease management consultations mean = 3.04, median = 0). As such each of these items was recoded into categorical variables to more accurately reflect these wide ranges. Analyses of sample characteristics were completed using descriptive statistics. 

The first objective to validate an adapted version of the OCP was analyzed using a principal component analysis (PCA), applying the guidelines outlined by Field (2009) [23]. A PCA was appropriate for this analysis because there is no way of knowing the number and kind of factors expected from this data. This analysis was followed by a reliability analysis, using Cronbach’s Alpha, to determine the internal consistency of responses. Finally, the factors were scored by determining the mean using scale responses for each item. 

The second objective to examine potential relationships between the cultural factors identified using the validated OCP and a number of socialization and education variables was measured using independent samples *t*-test for education and practice setting variables, and simple linear regression for years in practice [23]. The third objective to examine potential relationships between the cultural factors identified using the validated OCP and socialization and education variables was measured using multivariate ordinal regression, wherein the cultural factors were treated as the independent variables and pharmacists’ responses to the MTM services questions were treated as dependent variables. 

## 4. Results

A total of 303 US community pharmacists completed the survey, making the response rate 21%. Respondents had been in practice for an average of 23.4 years (SD = 12.1 years). Most respondents had completed a BSc in pharmacy (65%), and were working in a chain community pharmacy setting (65%). Compared to the wider community pharmacy population these respondents have been in practice longer, which accounts for the higher number of solely BSc Pharm trained respondents [27]. However, the breakdown of respondents working in chain versus independent pharmacies is consistent with the wider community pharmacy population [28,29]. The majority of respondents provided at least some advanced services to patients, with immunizations being the most frequently provided service (see Table 1 for complete details). 

*Objective 1. The PCA*: The initial PCA was conducted on the 40 item OCP with orthogonal rotation (varimax). An examination of the factorability using the Kaiser-Meyer-Olkin (KMO) measure verified the sampling adequacy for the analysis, KMO = 0.91, and all items for individual values were > 0.86, which is well above the standard limit of 0.5. Bartlett’s test of sphericity, which was *x*^2^ (300) = 4049.12, *p* < 0.0001, indicated that correlations between items were sufficiently large for a PCA. A total of 15 items were eliminated because they did not have a primary factor loading of 0.4, and/or no cross-loading of 0.3 or above. The final PCA was conducted on 25 items using the previous approach. The KMO measure verified the sampling adequacy for the analysis, KMO = 0.90, and all items for individual values were > 0.78, which is still well above the standard limit of 0.5. Bartlett’s test of sphericity, which was *x*^2^ (300) = 3043.33, *p* < 0.0001, indicated that correlations between items were sufficiently large for the PCA. An eigenvalue analysis identified 6 components with a score over 1 and a combination of 63% total explained variance. After an examination of the scree plot, consideration of the large sample size, and the Kaiser’s criterion, 6 factors were retained. 

The items that clustered on each factor suggest that factor 1 represents *social responsibility*, factor 2 represents *innovation*, factor 3 represents *people orientation*, factor 4 represents *competitiveness*, factor 5 represents *attention to detail*, and factor 6 represents *reward orientation*. The internal consistency for each of the factors was relatively high ranging from a Cronbach’s alpha of 0.73 for attention to detail to 0.82 for innovation. 

Composite scores of each of the factors were created calculating the mean of the items comprising the factor, with higher scores indicting that respondents perceived greater value in the factor. The highest score was on shown on the factor attention to detail (M = 4.16, SD = 0.68), and the lowest score was on the factor innovation (M = 3.10, SD = 0.73). As outlined in Table 2 all of the factors were negatively skewed, however, both the skewness and kurtosis were well within the tolerable ranges for assuming a normal distribution [23]. 

*Objective 2. Relationships between OCP factors and socialization/education variables*: The examination of the relationship between the socialization/education variables and the six OCP factors revealed a number of significant relationships. To begin a simple linear regression found a significant relationship predicting respondents’ valuing of the factor social responsibility based on pharmacists’ years in practice (YiP) (F(1, 286) = 9.64, *p* < 0.002, with an *R^2^* of 0.033). That is, respondents predicted value in social responsibility is equal to 3.43 + 0.01 (YiP) points when it is measured in the OCP. 

Next independent sample *t*-tests were conducted to examine the relationships between levels of pharmacists’ education (BSc Pharm vs. PharmD), and practice setting (Chain vs. Independent), and the OCP factors. Pharmacists with BSc Pharmacy training perceived greater value in the factor people orientation (M = 3.56, SE = 0.055), than pharmacists with PharmD training (M = 3.36, SE = 0.07). However, pharmacists with PharmD training perceived greater value in the factor attention to detail (M = 4.27, SE = 0.07), than pharmacists with BSc Pharmacy training (M = 4.10, SE = 0.05). Pharmacists practicing in independent pharmacies perceived greater value in social responsibility (M = 3.88, SE = 0.05), than pharmacists working in chain pharmacy settings (M = 3.57, SE = 0.05). Additionally, pharmacists working in independent pharmacies also perceived greater value in people orientation (M = 3.65, SE = 0.08), than pharmacists working in chain pharmacy settings (M = 3.41, SE = 0.05). See Table 3 for complete results. 

*Objective 3. Relationships between OCP factors and MTM service provision*: As first step to exploring objective three, ordinal regression models were run with each of the socialization/education variables to determine if confounding was possible. The only significant relationship noted here was that pharmacists from the chain community pharmacy setting were more likely to provide a greater number of immunizations per month, than pharmacists working in an independent community pharmacy setting (OR 7.24, 95% CI 4.02–13.03). However, all of the socialization/education variables were used in subsequent modeling.

The adjusted ordinal regression models for the OCP factors and immunizations, medication reviews, and disease state consultations, identified a number of significant relationships (see Table 4). More specifically, those respondents who perceived value in the factors social responsibility, innovation, and competitiveness were more likely to provide immunizations than those respondents who perceived less value in these factors. Respondents who perceived value in the factor innovation were more likely to provide a higher number of medication reviews per month (OR 1.92, 95% CI 1.40–2.65). However, respondents who perceived value in the factor attention to detail were less likely to provide a higher number of medication reviews per month (OR 0.67, 95% CI 0.47–0.94). Finally, only innovation was significantly associated with disease state consultations. In particular, those respondents who perceived value in innovation were more likely to provide disease state consultations (OR 1.72, 95% CI 1.25–2.46). 

## 5. Discussion

This work provides some preliminary insights into the professional culture of community pharmacy in the US using the OCP, and how it may influence community pharmacists’ provision of MTM services. The study identified six distinct cultural factors including: social responsibility, innovation, people orientation, competitiveness, attention to detail, and reward orientation. Respondents to the survey saw the greatest value in the factor attention to detail. When compared to previous Canadian data, the sample of US pharmacists also saw greater value in social responsibility and competitiveness, and also identified two new factors people orientation and attention to detail [21].

The regression models found that those respondents seeing greater value in the factors social responsibility, innovation, and competitiveness provided a higher number of immunizations. Furthermore, those respondents seeing greater value in innovation also provided more medication reviews and disease state consultations. When compared to Canadian data, a similarly positive relationship was noted for the factors competitiveness and innovation, and the provision of advanced pharmacy services [21]. Moreover, two other studies conducted in the US, one examining organizational factors influencing pharmacy practice change, and another examining influence on pharmacy service provision, found that entrepreneurial orientation and innovation were both positive influences on the provision of advanced pharmacy services [30].

Contrarily, respondents from this survey who saw greater value in attention to detail provided fewer medication reviews. This inverse relationship was unique to the US sample of community pharmacists. It is also worth noting that when compared to BScPharm trained respondents, PharmD trained respondents also saw greater value in this factor. A partial explanation for this emphasis within the PharmD trained population may be a function of the increased attention to the mitigation of medication errors that had taken place in the profession when transitioning to this training model [31]. It is also important to recognize that in and of itself attention to detail is not a negative cultural factor, as it likely contributes to pharmacists’ ability to accurately fill patients’ prescriptions. Rather it may be influenced by other contextual factors within the community pharmacy environment. For example, time constraints, as noted in other research, might result in pharmacists’ dedicating the limited time they have to ensuring that patients’ prescriptions are correct [7]. 

Taken together, all of these findings show an important overlap between social responsibility and competitiveness, which were also identified by the majority of survey respondents as being important, and the provision of immunizations to patients. A recent study found that from 2007 to 2013 the number of influenza vaccinations dispensed in community pharmacies increased from 3.2 to 20.9 million, showing that the respondents’ perceptions of these factors, and self-reported provision of immunizations, are likely based on the actual care of patients [32]. 

However, the inverse relationship between attention to detail and the number of medication reviews provided, and that innovation was seen to be less important by most respondents, though connected with the higher provision of both medication reviews and disease state consultations, is potentially problematic. If the profession wishes to see the continued expansion of its scope of practice, and payment for these services, ensuring that the majority of pharmacists are actively providing these services is essential. 

There are a number of ways in which innovation could be fostered in community pharmacists. To begin current community pharmacists and pharmacy students could be provided skills through coursework encouraging innovative thinking for problem resolution, reflective thinking, risk taking, and adaptability. In fact, this is training is already taking place through programs like Educating Pharmacists in Quality (EPIQ), which was developed and offered free of charge by the Pharmacy Quality Alliance [33]. As part of this program students learn how to “measure, improve, and report quality of care” [33]. However, perhaps a more explicit integration of the items comprising the factor innovation is possible?

It is also important to recognize that the reach of these programs is not complete. As such, this research should be extended and developed to apply the findings from this study to determine how to best help pharmacists transition their practices. This research, will go beyond simply mandating pharmacists to be more innovative. Rather it will be of key importance to account for the entire context in which pharmacists practice, as advocated for by implementation research [9,10]. A systematic review of implementation research studies in allied health professions identified 12 pharmacy-specific before-after and cohort studies, which primarily provided educational material to facilitate the integration of evidence into practice [34]. In addition to being of low methodological quality, these studies also make the underlying assumption that community pharmacists do not implement evidence because they lack appropriate education [34]. While this may be an important component of non-implementation, these studies have failed to account for structural barriers including poor workflow and staffing management that prevent pharmacists from taking adequate time to interact directly with patients in need of further intervention. 

Two examples of studies furthering the objective of more fully accounting for the entire context of community pharmacy practice include: a qualitative study examining community pharmacists’ perceptions of barriers and facilitators to targeted organizational structure adaptations to enhance the provision of medication adherence programming, and a cluster randomized trial to employ a facilitation intervention to improve the provision of medication management services in the community setting [35,36]. Moreover, the cluster randomized trial observed a decrease in the number of medication management services offered during the influenza vaccination season [36]. This means that future studies need to consider not only the time of year when interventions are implemented, but also consider how to deal with patients in need of medication reviews during this time period. 

*Study limitations*: There are a few limitations to this study. First the use of the healthcare research firm to administer the survey may have consequences with respect to the generalizability of the survey results. However, given the correlations with previous Canadian findings it seems reasonable to suggest that this sample is not completely out of sync with the community pharmacy population in the US. Second, this work makes the assumption that at least part of the decision to provide MTM services rests with the pharmacist [21]. Without question, there are structural issues like high dispensing volumes, which were not accounted for in this study, that make it difficult for pharmacists to take on new practice opportunities, but this influence is not complete [6]. Third, MTM does not encompass all clinical services that community pharmacists may provide. As such, it is possible that possible relationships between the OCP and clinical services were missed. Fourth, while the sample of respondents from the southeastern US was randomly selected the characteristics of non-responders was not tracked. As such, the generalization of these findings to all community pharmacists should be approached with caution. Finally, the OCP provides just one perspective on the professional culture of community pharmacists in the US. To obtain a more complete vision of this professional culture additional work will need to be completed in this area. 

## 6. Conclusions

The professional culture of pharmacy is an important part of the context of community pharmacy practice, of which a better understanding is required to ensure the successful integration of new practice opportunities into this setting. This validation of the OCP in a sample of community pharmacists from the US identified 6 cultural factors: social responsibility, innovation, people orientation, competitiveness, attention to detail, and reward orientation. A number of significant relationships were also observed between the factors and pharmacists’ self-reported provision of MTM services like immunizations. This knowledge about the professional culture of community pharmacists provides further insight into the context of this environment, which when added to knowledge of structural barriers will improve pharmacists’ ability to successfully implement new pharmacy services into their practices.

## Figures and Tables

**Table 1:** Respondent characteristics.

		Proportion (Frequency)
Highest level of education	BSc Pharm	65% (190)
	PharmD	35% (105)
	MSc Pharm	1% (4)
	Pharmacy residency	0.7% (2)
	Missing	0.3% (1)
Practice setting	Community pharmacy chain store	65% (195)
	Community pharmacy independent store	34% (104)
	Missing	1% (3)
Services	Immunizations	
	0	20% (61)
	1–20	59% (179)
	21<	20% (61)
	Missing	0.3% (1)
	Medication reviews	
	0	32% (96)
	1−10	49% (149)
	11−51<	17% (51)
	Missing	2% (6)
	Disease management consultations	
	0	58% (176)
	1−10	34% (104)
	11<	6% (18)
	Missing	1% (4)

**Table 2:** Descriptive statistics for the 6 OCP factors (*N* = 300).

	Factor Loadings	Mean (SD)	Skewness (SE)	Kurtosis (SE)	Cronbach’s α
**Social responsibility**		3.67 (0.71)	−0.23 (0.14)	−0.19 (0.28)	00.81
Having a good reputation	0.74	4.11 (0.85)			
An emphasis on quality	0.71	4.09 (0.88)			
Being socially responsible	0.67	3.63 (0.92)			
Being calm	0.60	3.14 (1.1)			
Developing friends at work	0.60	3.42 (0.96)			
**Innovation**		3.10 (0.73)	0.25 (0.14)	−0.16 (0.28)	0.82
Being innovative	0.78	3.42 (0.96)			
Being reflective	0.73	3.20 (0.87)			
Being quick to take advantage of opportunities	0.71	3.28 (1.0)			
Adaptability	0.68	3.37 (0.95)			
Risk taking	0.61	2.71 (1.0)			
**People orientation**		3.49 (0.75)	−0.13 (0.14)	−0.28 (0.28)	0.77
Tolerance	0.74	3.40 (0.90)			
Informality	0.74	3.06 (1.0)			
Fairness	0.64	3.69 (0.98)			
Taking individual responsibility	0.54	3.78 (0.99)			
**Competitiveness**		3.67 (0.69)	−0.19 (0.14)	−0.21 (0.29)	0.76
Being results oriented	0.73	3.94 (0.91)			
Being aggressive	0.73	3.22 (1.0)			
Achievement orientation	0.63	3.65 (0.95)			
Being competitive	0.57	3.91 (0.92)			
Confronting conflict directly	0.44	3.61 (0.97)			
**Attention to detail**		4.16 (0.68)	−0.59 (0.14)	−0.35 (0.28)	0.73
Being rule oriented	0.81	4.24 (0.85)			
Paying attention to detail	0.71	4.39 (0.79)			
Being analytical	0.70	3.84 (0.88)			
**Reward orientation**		3.20 (0.85)	−0.06 (0.14)	−0.11 (0.28)	0.75
Security of employment	0.76	3.50 (0.97)			
High pay for good performance	0.75	3.08 (1.1)			
Offers of praise for good performance	0.64	3.01 (1.1)			

SD = Standard deviation, SE = Standard error; Scale: from 1 not at all recognized to 5 very much recognized.

**Table 3:** Independent sample *t*-tests between level of education and practice setting, and OCP factors.

	BSc Pharm N = 190	PharmD ^ N = 105
**Cultural factors**	Mean (SE)	Mean (SE)
Social responsibility	3.76 (0.05)	3.53 (0.07)
Innovation	3.20 (0.05)	3.17 (0.07)
People orientation	3.56 (0.06) *	3.35 (0.07) *
Competitiveness	3.69 (0.05)	3.64 (0.07)
Attention to detail	4.10 (0.05) *	4.27 (0.07) *
Reward orientation	3.20 (0.06)	3.20 (0.09)
	***Chain N = 195***	***Independent N = 104***
**Cultural factors**	Mean (SE)	Mean (SE)
Social responsibility	3.57 (0.05) ***	3.88 (0.07) ***
Innovation	3.17 (0.05)	3.24 (0.08)
People orientation	3.41 (0.05) **	3.65 (0.08) **
Competitiveness	3.73 (0.05)	3.58 (0.07)
Attention to detail	4.17 (0.05)	4.14 (0.07)
Reward orientation	3.17 (0.06)	3.26 (0.09)

* *p* < 0.05, ** *p* < 0.01, *** *p* < 0.001; SE = Standard error; Scale: From 1 not at all recognized to 5 very much recognized; ^ Only those respondents with either a BSc Pharm or a PharmD were included in this analysis.

**Table 4:** Results of multivariate ordinal regression of pharmacy services and OCP factors.

Service ^ (DV)	OCP Factor (IV)	Odds Ratio *	CI (95%)
Immunizations	Practice setting	7.24	4.02–13.03
	Social responsibility	1.46	1.03–2.06
	Innovation	1.58	1.14–2.21
	Competitiveness	1.75	1.22–2.52
Medication reviews	Innovation	1.92	1.40–2.65
	Attention to detail	0.67	0.47–0.94
Disease state consultations	Innovation	1.75	1.25–2.46

^ All services integrated into the models as previously developed categorical variables; * Only statistically significant odds ratios presented in this table; DV = dependent variable; IV = independent variable.

## Supplementary Materials

The following materials are available online at http://www.mdpi.com/2226-4787/6/2/25/s1.
