# Supplementary material for: The Professional Culture of Community Pharmacy and the Provision of MTM Services
Source: Pharmacy (Basel). 2018 Mar 21;6(2):25. doi: 10.3390/pharmacy6020025 (PMC6025126; doi:10.3390/pharmacy6020025)
Supplement: Supplementary file 1 [file pharmacy-06-00025-s001.pdf]

Professional culture survey

By checking this box I certify that I am 18 years of age or older

○

Investigator:
Meagen Rosenthal, PhD
Department of Pharmacy Administration
University of Mississippi
mmrosent@olemiss.edu
(662) 915-2475

Description:
The purpose of this research project is to gain insight into the professional culture of pharmacy, and how that culture may influence pharmacists' decisions to provide additional clinical services to their patients. We would like to ask you a few questions about your ideas about pharmacists' professional culture, and your intention to provide clinical services to patients. You will not be asked for your name, or any other identifying information.

Risks and Benefits:
You may feel uncomfortable with the length of time needed to complete the survey. We do not thing that there are any other risks.

Confidentiality:
No identifiable information will be recorded, therefore, we do not think that you can be identified from this study.

Right to Withdraw:
You do not have to take part in this study and you may stop participation at any time up until you submit the survey. You may skip any questions you prefer not to answer. However, please note once you have submitted the survey we have no way of removing your responses, because we are not collecting any identifying information. If you have any questions regarding this please contact Dr. Meagen Rosenthal (contact information above).

IRB Approval:
This study has been reviewed by the University of Mississippi's Institutional Review Board (IRB). The IRB has determined that this study fulfills the human research subject protections obligations required by state and federal law and University policies. If you have any questions, concerns, or reports regarding your rights as a participant of research, please contact the IRB at (662) 915-7482.

Statement of Consent:
I have read and understand the above information. By completing the survey I consent to participate in the study.

Background questions

In what year did you become a licensed pharmacist?

[ ]

Please indicate your educational achievements (please check all that apply):

- BSc Pharm
- Pharm D
- MSc Pharm
- Pharmacy Residency

Thinking about your student training please rank from 1 (most) to 5 (least) the time you spent in each setting:

- Community pharmacy chain store
- Community pharmacy independent store
- Ambulatory pharmacy
- Institutional pharmacy
- Specialty pharmacy

Please indicate your current pharmacy practice setting:

- Community pharmacy chain store
- Community pharmacy independent store

In the past month how many medication reviews have you completed?

If you haven't provided any medication reviews in the past month, please select the most significant barrier to providing medication reviews?

- Time - my store is too busy to spend the required time with patients
- Training - I would need further education before I could confidently conduct medication reviews
- Patients - my patients are not interested in receiving this kind of service
- Organization - my organization doesn't support providing medication reviews

In the past month how many immunizations have you provided?

If you haven't provided any immunizations in the past month, please select the most significant barrier to providing immunizations?

Season - we only provide flu vaccinations

- Time - my store is too busy to spend the required time with patients
- Training - I would need further education before I could confidently provide immunizations
- Patients - my patients are not interested in receiving this kind of service
- Organization - my organization doesn't support providing immunizations

In the past month how many disease management consultations have you provided?

If you haven't provided any disease management consultations in the past month, please select the most significant barrier to providing disease management consultations?

- Time - my store is too busy to spend the required time with patients
- Training - I would need further education before I could confidently provide disease management consultations
- Patients - my patients are not interested in receiving this kind of service
- Organization - my organization doesn't support providing disease management consultations

Professional culture:

In this section we are interested in your perception of the profession of pharmacy. For each of the following items please rate,

"To what extent is pharmacy recognized for its..."

	Not at all	Minimally	Moderately	Considerably	Very much
**Adaptability**					
**Stability**					
**Being reflective**					
**Being innovative**					
**Being quick to take advantage of opportunities**					
**Taking individual responsibility**					
**Risk taking**					
**Opportunities for professional growth**					
	Not at all	Minimally	Moderately	Considerably	Very much
**Autonomy**					
**Being rule oriented**					

	Not at all	Minimally	Moderately	Considerably	Very much
**Being analytical**					
**Paying attention to detail**					
**Confronting conflict directly**					
**Being team oriented**					
**Sharing information freely**					
**Being people oriented**					
	Not at all	Minimally	Moderately	Considerably	Very much
**Fairness**					
**Not being constrained by many rules**					
**Tolerance**					
**Informality**					
**Decisiveness**					
**Being competitive**					
**Being highly organized**					
**Achievement orientation**					
	Not at all	Minimally	Moderately	Considerably	Very much
**Having a clear guiding philosophy**					
**Being results oriented**					
**Having high performance expectations**					
Being aggressive					
**High pay for good performance**					
**Security of employment**					
**Offers of praise for good performance**					
**Being supportive**					
	Not at all	Minimally	Moderately	Considerably	Very much
**Being calm**					
**Developing friends at work**					
**Being socially responsible**					
**Enthusiasm for the job**					
**Working long hours**					
**Having a good reputation**					
**An emphasis on quality**					
**Being distinctive/different from others**					
